# DNA transducer-triggered signal switch for visual colorimetric bioanalysis

**DOI:** 10.1038/srep11190

**Published:** 2015-06-10

**Authors:** Wenhong Chen, Yurong Yan, Ye Zhang, Xuemei Zhang, Yibing Yin, Shijia Ding

**Affiliations:** 1 Ministry of Education Key Laboratory of Child Development and Disorders; Key Laboratory of Pediatrics in Chongqing, CSTC2009CA5002; Chongqing International Science and Technology Cooperation Center for Child Development and Disorders; Department of Clinical laboratory, Children’s Hospital of Chongqing Medical University, Chongqing 400014, PR China; 2 Key Laboratory of Clinical Laboratory Diagnostics (Ministry of Education), College of Laboratory Medicine, Chongqing Medical University, Chongqing 400016, PR China

## Abstract

A simple and versatile colorimetric biosensor has been developed for sensitive and specific detection of a wide range of biomolecules, such as oligonucleotides and aptamer-recognized targets. Combining the signal transducer and catalyzed hairpin assembly (CHA)-based signal amplification, the target DNA binds with the hairpin DNA to form a new nucleic acid sequence and creates a toehold in the transducer for initiating the recycle amplification reaction of CHA. The catalyzed assembly process produces a large amount of G-rich DNA. In the presence of hemin, the G-rich DNA forms G-quadruplex/hemin complex and mimic horseradish peroxidase activity, which catalyzes a colorimetric reaction. Under optimal conditions, the calibration curve of synthetic target DNA has good linearity from 50 pM to 200 nM with a detection limit of 32 pM. This strategy has been successfully applied to detect *S. pneumoniae* as low as 156 CFU mL^−1^, and shows a good specificity against closely related *streptococci* and major pathogenic bacteria. In addition, the developed method enables successful visual analysis of *S. pneumoniae* in clinical samples by the naked eye. Importantly, this method demonstrates excellent assay versatility for sensitively detecting oligonucleotides or aptamer-recognized targets.

*Streptococcus pneumoniae*, one of the most common bacterial respiratory pathogens worldwide, causes several infectious diseases including community acquired pneumonia, otitis media, meningitis and septicemia^1^,^2^. In the developing world, pneumococcal pneumonia leads to 25% of all preventable deaths in children under 5 years old and over 1.2 million infant deaths every year^3^,^4^. Unfortunately, the reported incidence rates for laboratory confirmed invasive pneumococcal disease underestimate the true burden, as only a small portion of presumptive cases can be confirmed by conventional techniques.

Numerous analytical methods have been developed for detection of *S. pneumoniae*. Conventional culture method is reliable, but time-consuming, and it sometimes compromises by spontaneous autolysis or antibiotic treatment. In addition, direct pneumococcal antigen detection, including ELISA and a rapid immunochromatographic test (Binax NOW *S. pneumoniae* urinary antigen test), has a poor sensitivity and specificity. Moreover, urine antigen-based testing can be positive for weeks after the onset of infection^5^,^6^. Polymerase chain reaction (PCR) frequently applied is independent of bacterium viability and has distinct advantage in sensitivity^7^. But PCR wide application has been limited by its false positivity and the low resolution of post-PCR analysis by gel electrophoresis^8^. Real-time quantitative PCR can provide a higher sensitivity^9^. However, it requires sophisticated, expensive equipments and highly trained analysts in a well maintained laboratory^10^. Compared with these methods, colorimetric sensors have recently attracted special attention owing to their extreme simplicity and the visual detection in homogeneous solution without any immobilization, separation and washing steps. In this study, colorimetric detection is based on repetitive guanine rich sequence motifs, which can form G-quadruplex/hemin complex (DNAzyme) in the presence of hemin. DNAzyme, having the mimic horseradish peroxidase activity^9^,^11^,^12^, possesses higher thermal stability that can be denatured and renatured for many cycles without losing catalytic activities. Thus, DNAzyme can catalyze the conversion of a colorless 2, 2′-azino-bis (3-ethylbenzothiazoline-6-sulfonic acid) (ABTS^2−^) to a green ABTS^−^13.

Signal amplification is of importance for molecular detection. To date, a variety of amplification strategies have been applied to further enhance the sensitivity. Most of them need the participation of enzyme^14^,^15^, label^16^, or materials^17^,^18^, with the drawbacks including specific reaction conditions, exorbitant price, poor stability, complicated operation and so on. Recently, catalytic hairpin assembly, developed from DNA nanostructure organization, has been received particular interest. CHA relies only on hybridization and strand-exchange reactions to achieve signal amplification circuit^19^,^20^,^21^, which overcomes the weaknesses of enzymatic amplification and the utilization of materials^22^,^23^. However, CHA currently has the disadvantage of requiring complicated engineering for adaptation to various analytes^24^. Redesigning the entire circuit each time is unnecessarily arduous for the clinical analytical applications.

Aim to develop a versatile colorimetric biosensor by reusing the circuit’s module to detect various inputs through the use of a transducer. In this study, a novel colorimetric sensing strategy by combining the signal transducer and CHA is developed for detecting the part of *lytA* gene of *S. pneumoniae*^25^,^26^ and the whole cell of *Salmonella Typhimurium*^27^. The method reveals a great potential to adapt CHA to support even more robust analytical applications, and construct a label-free, enzyme-free, visual, simple, rapid, sensitive and specific platform for the development of low-cost and point-of-care diagnostics. It may be a potential and powerful tool for clinical diagnostics in the future.

## Results and Discussion

### Design of the proposed method

**Figure 1:**
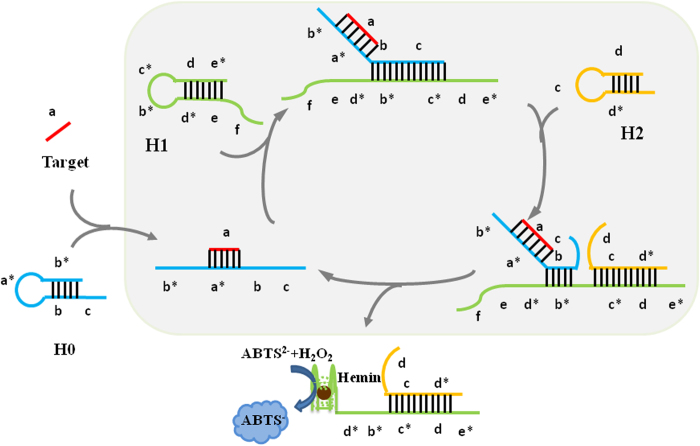
A schematic illustration of the signal transducer and CHA-based circuit for detecting the *lytA* gene of *S. pneumonia.*

The principle of the proposed method is illustrated in Fig. 1. The system consists of the signal transducer, CHA amplification and DNAzyme signal readout steps. The target T0 (in red) is the part of lytA gene of S. pneumoniae. Domain b and c of the hairpin H0 (in blue), have been designed according to the sequences of H1, serve as a toehold for initiating the amplification reaction. The signal transducer H0 is generated by extending the 3’-end of domain b and c to form a hairpin loop where domain b* of H0 is occluded by domain b. Therefore H0 should be catalytically inactive. The loop a* is the complementary sequence to 24 nt of *lytA* gene nucleic acid. A pair of hairpins was designed based on our previous research works^28^. The two hairpins (H1 and H2) do not initially interact with each other but can catalytically form a duplex in the presence of unstructured domain b and c. The unstructured domain e and f of H1 are able to form G-quadruplex. When domain e is hidden in the stem of H1, the G-quadruplex cannot be formed at this time. In the presence of target, the loop can hybridize with the target, forming a rigid duplex and disrupting the stem formed by domain b*and b. The liberated b and c further open H1, leading to the formation of toehold for strand displacement by H2. Then H2 hybridizes with the unfolded H1 and releases the complex of T0 and H0 (T0-H0) into next circulation. As a result, the generated abundant G-quadruplex structures are proportional to targets. The G-quadruplex structure can strongly bind with hemin to form the DNAzyme, displaying peroxidase-like activity, catalyzing oxidation of ABTS^2−^ to ABTS^−^.

### Feasibility of proposed method

**Figure 2:**
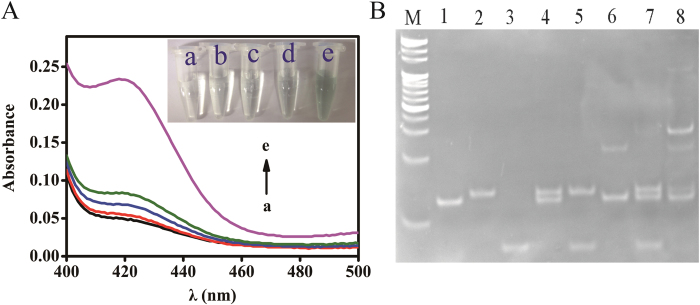
(**A**) Absorption spectra of sample solutions under different conditions: (a) H1; (b) H1+H2; (c) H0+H1+H2; (d) T0+H0+H1; and (e) T0+H0+H1+H2. Inset: The corresponding photographs of the colorimetric responses. (**B**) PAGE of sample solutions under different conditions: M: 20-bp marker; 1: H0; 2: H1; 3: H2; 4: H0+H1; 5: H1+H2; 6: T0+H0; 7: H0+H1+H2; 8: T0+H0+H1+H2.

To validate whether the detectable signal really derived from the target or not, the comparative experiments were performed to prove feasibility of the proposed method. After incubation with hemin for 40 min, the absorbance of different mixtures was recorded. As shown in Fig. 2A, the system containing H1 exhibited very weak characteristic absorption peak at 418 nm (Fig. 2A, curve a). Compared with curve a, there was almost no change in the mixture of H1 and H2 (Fig. 2A, curve b). Upon the addition of H0 to the H1, H2 system, a slight increase in absorption peak was detected (Fig. 2A, curve c). The results demonstrated that H0 can trigger weak interaction of H1 and H2 to form a small amount of self-assembly product, resulting in background signal. When the mixture of 500 pM T0, H0 and H1 was incubated together, the absorbance increased slightly (Fig. 2A, curve d), indicating that T0 can trigger the self-assembly of H0 and H1, forming a certain amount of G-quadruplex structures. But in the presence of T0 and H2 (Fig. 2A, curve e), the amplified signal was about 282% of that without H2 (Fig. 2A, curve d). Such significant signal enhancement clearly indicates that the cycling amplification strategy can be executed with the aid of hairpin H2. The insets of Fig. 2A show the corresponding photographs of the colorimetric responses.

We also used native polyacrylamide gel electrophoresis (PAGE) to characterize the reaction process (Fig. 2B). The base number of H0 (lane 1), H1 (lane 2) and H2 (lane 3) derived from PAGE were in accordance with our design. Significantly, the mixture of H0 and H1 did not cause their self-hybridization reaction (lane 4), neither did the mixture of H1 and H2 (lane 5). T0 hybridized with the partial H0 (lane 6). When we mixed the H0, H1 and H2, there was a barely visual band (lane 7) above H0, H1 and H2, which was H1-H2 complex. Expectedly, adding T0 to the H0 solution followed by incubation with H1 and H2, the H1-H2 band (lane 8) became much brighter compared with H1-H2 of lane 7.

### Optimization of the reaction conditions

**Figure 3:**
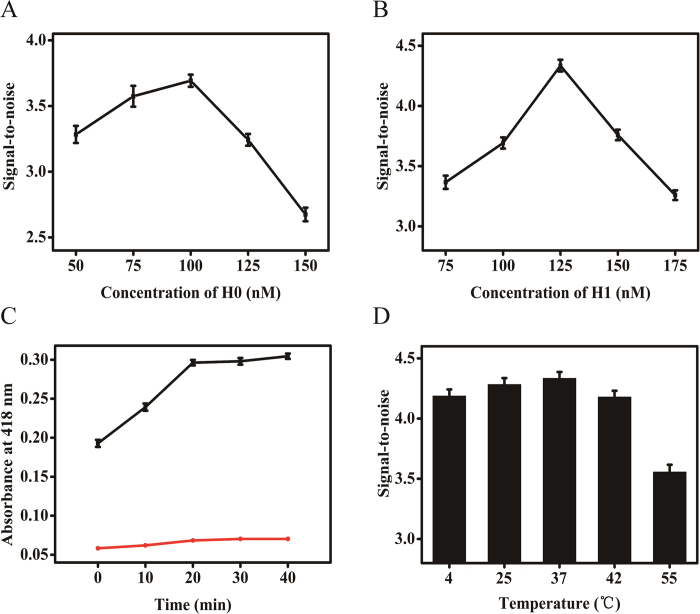
Effects of (**A**) H0 concentration on signal-to-noise ratio, (**B**) H1 concentration on signal-to-noise ratio, (**C**) dependence of H1/H2 on incubation time in the presence of target T0 1 nM (black), and further absence of target T0 (red), (**D**) incubation temperature on the H1/H2. The error bars were standard deviations of three parallel experiments.

To achieve the optimal analytical performance, different reaction conditions were optimized. First, the effect of concentration of hairpin H0 and H1 were investigated respectively. High concentration of hairpin structures can increase the self-assembly, but correspondingly increase the noise owing to the potential hybridization of hairpins. To avoid the signal increase induced by nonspecific background amplification, the signal-to-noise ratio is used to evaluate the sensitivity of the assay. The maximum value of signal-to-noise ratio was achieved at the H0 concentration of 100 nM (Fig. 3A). The concentration of H1 was also critical parameter affecting the analytical performance. As showed in Fig. 3B, 125 nM H1 was chosen for the subsequent experiments according to the maximum signal-to-noise ratio.

Time dependence of the T0-H0 catalyzed hairpin assembly was also investigated. As shown in Fig. 3C, in the absence of target T0, only very weak signal increase was observed (in red). In the presence of target T0 (in black), with the increase of the incubated time, the signal synchronously ascended. The continuously increasing signal also indicated that the T0-H0 catalyzed hairpin assembly was indeed taking place. Beyond the incubation time of 20 min, the absorbance intensity reached a stable value. Therefore, 20 min was selected as the optimal incubation time for CHA. Finally, the incubating temperature was examined from 4 to 55 °C (Fig. 3D). At 37 °C, the signal-to-noise ratio reached the top, so 37 °C was selected as the appropriate temperature.

### Analytical performance of colorimetric biosensor

**Figure 4:**
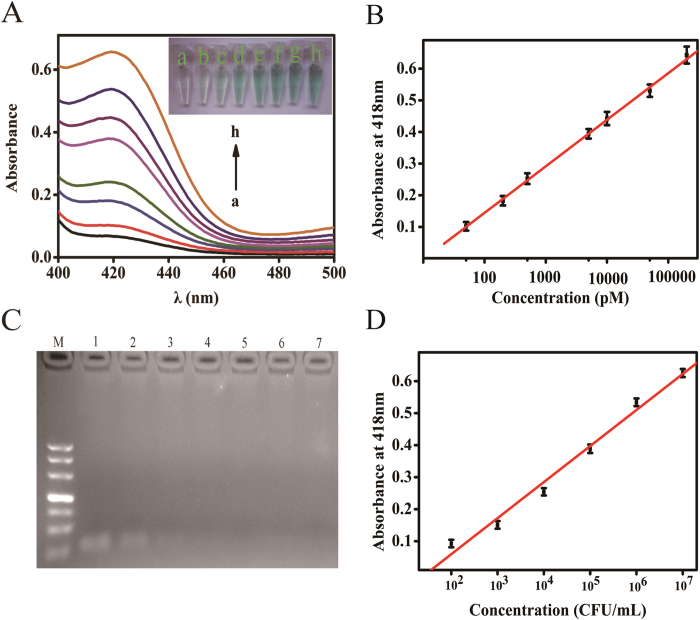
Sensitivity investigation by detecting targets at various concentrations: (a) 0 pM; (b) 50 pM; (c) 200 pM; (d) 500 pM; (e) 5 nM; (f) 10 nM; (g) 50 nM; (h) 200 nM. (**A**) UV–vis absorption spectra in a region of 400–500 nm. Inset: The corresponding photographs of the colorimetric responses. (**B**) Relationship of absorption peak with logarithm of the target T0 concentration. (**C**) Agarose gel electrophoresis of *S. pneumoniae 19F* PCR products. (1) 10^7^, (2) 10^6^, (3) 10^5^, (4) 10^4^, (5) 10^3^, (6) 10^2^ CFU mL^−1^ *S. pneumoniae 19F* and (7) blank. (**D**) Relationship of absorption peak with logarithm of the *Streptococcus pneumoniae 19F* concentration. The error bars were standard deviations of six repetitive measurements.

Under the optimal experimental conditions, the UV−vis absorption increase with the increasing concentration of target T0. As shown in Fig. 4A, the absorbance intensity enhanced successively with the increasing concentration of target DNA. As expected, the inset showed the color variation of the reaction solution for the increasing amounts of T0, a series of noticeable color change could be visualized from light to deep (inset of Fig. 4A). By analyzing the absorbance with the concentrations of T0 (Fig. 4B), the linear curve of the absorbance intensity (Y) vs the logarithm of the T0 concentration (C) was fitted to a regression equation of Y = 0.1471 lg (C/pM) - 0.1498 (R^2^ = 0.9994) in the range of 50 pM–200 nM. The detection limit was calculated to be 32 pM according to the responses of the blank tests plus 3 times the standard deviation. The relative standard deviations (RSD) of the intra-assay and inter-assay precisions were 3.6% and 5.9%, respectively. These results demonstrate that the developed colorimetric assay has good precision and acceptable reproducibility.

To evaluate the reliability and practicability of the proposed method, the detection of the PCR products using the developed method was carried out by culturing *S. pneumoniae* at the concentrations from 0 to 2.4 × 10^7^ CFU mL^−1^. After a pretreatment step for the samples, PCR was performed using target *lytA* gene extracted from each concentration of *S. pneumoniae*, and PCR products were verified by agarose gel electrophoresis (Fig. 4C). There was no target band of PCR products for *S. pneumoniae* less than 2.4 × 10^5^ CFU mL^−1^ owing to the low resolution of gel electrophoresis^29^. Meanwhile, the denatured PCR products were also detected using the developed colorimetric biosensor. As shown in Fig. 4D, a good linear dependence on absorbance intensity with logarithm of the *S. pneumoniae* concentration was achieved in the range from 10^2^ to 10^7^ CFU mL^−1^. The calibration curve can be expressed as Y = 0.1126 lg(C/pM) –0.1659 (R^2^ = 0.9926), and the detection limit was calculated to be 156 CFU mL^−1^ (n = 3), which is much lower than previous reported methods (Supplementary Table S1). The relative standard deviations (RSD) of the intra-assay and inter-assay precisions were 4.5% and 6.2%, respectively, demonstrating potential promise in clinical application.

### Specificity

**Figure 5:**
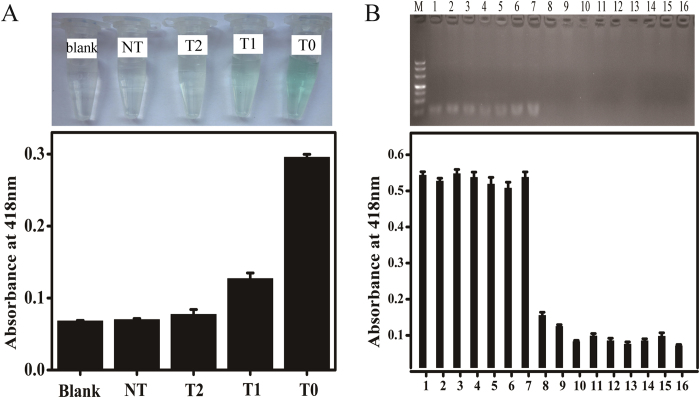
(**A**) Specificity of the assay for DNA detection: complementary target DNA (T0), single-base mismatched DNA (T1), two-bases mismatched DNA (T2), non-complementary DNA (NT), and blank. Inset: The corresponding photographs of the colorimetric responses. (**B**) Selectivity of the assay for bacteria detection: *S. pneumonia D39* (1), *S. pneumonia 19F* (2), *S. pneumoniae R6* (3), *S. pneumoniae 6B* (4), *S. pneumoniae TIGR4* (5), *S. pneumoniae TIGR3* (6), *S. pneumoniae 49619* (7), *S. mitis* (8), *Streptococcus pyogenes* (9), *Haemophilus influenza* (10), *Staphylococcus aureus* (11), *Enterococcus faecalis* (12), *Klebsiella pneumoniae* (13), *E.coli* (14), *Pseudomonas aeruginosa* (15), and blank (16). Inset: Agarose gel electrophoresis of different bacterial PCR products. The error bars were standard deviations of three repetitive measurements.

The specificity of the method was further investigated by exposing hairpins to a series of artificial targets sequences, including perfect complementary target DNA T0, single-base mismatched DNA (T1), two-bases mismatched DNA (T2), and non-complementary DNA (NT) at the same concentration (1 nM). As shown in Fig. 5A, the absorbance for one-base mismatched DNA and two bases mismatched DNA was only about 25.8% and 4.0% of that for perfect target, respectively. The non-complementary target DNA showed almost the same response as the blank solution, indicating high specificity and great potential for single nucleotide polymorphism analysis. This may be attributed to the relatively long stem region of the hairpin structure with thermodynamic stability. In addition, the specificity of the developed method was further explored using other streptococci and major pathogenic bacteria presented in clinical samples. As shown in Fig. 5B, *S. pneumoniae* and other bacteria at two different concentration levels (10^7^ and 10^8^ CFU mL^−1^) were analyzed. The results show that 7 strains *S. pneumoniae* have similar high signal responses among all the bacteria tested. The signal responses of other bacteria show no difference to that of the blank solution. Thus, this method displays excellent specificity and conservative for the detection of *S. pneumoniae*.

### Clinical sample assay

The potential application of the developed colorimetric biosensor was investigated for detection of *S. pneumoniae* in clinical samples. As shown in Supplementary Table S2, there were good correlation for the assaying results of clinical human bronchoalveolar lavage fluid samples and cerebrospinal fluid samples using and bacterial culture method, indicating the feasibility and acceptable accuracy of proposed method for detection of *S. pneumoniae* in complex clinical samples. In addition, the assaying values of the proposed method were slightly larger than bacterial culture plating count. These results may be attributed to detected DNA from dead bacteria. In the future research, a large number of clinical samples will be applied to further verify the developed method.

### Universality

**Figure 6:**
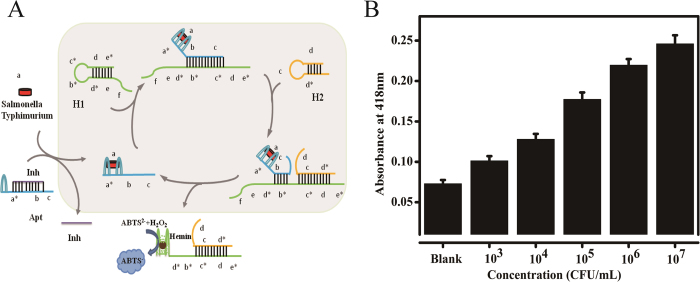
(**A**) Schematic diagram of the anti-*Salmonella Typhimurium* aptamer based CHA colorimetric detection (**B**) The peak of absorbance responding to serial dilutions of *Salmonella Typhimurium* in the range of 2 × 10^3^−2 × 10^7^ CFU mL^−1^. The error bars were standard deviations of three repetitive measurements.

In principle, non-nucleic acid can also be employed as inputs. Previous studies^24^,^30^ have shown that the aptamer can be used in conjugation with catalyzed hairpin assembly for the detection of non-nucleic-acid analytes. Therefore, based on the developed colorimetric strategy, *Salmonella Typhimurium* has been successfully detected using transducer with the similar circuit’s module. The principle of detecting *Salmonella Typhimurium* is illustrated in Fig. 6A. We design an aptamer beacon in which an anti-*Salmonella Typhimurium* aptamer^27^, Apt, is inhibited by a complementary strand, Inh. Apt contains the toehold (domain b and c) for initiating the amplification reaction, and Inh can block the catalyst region. When Apt is bound with Inh, it can neither bind *Salmonella Typhimurium* nor catalyze the hybridization of H1 and H2. In contrast, free Apt can do both. As a result, when the *Salmonella Typhimurium* binds to and stabilizes Apt, it shifts the equilibrium so that the duplex with Inh is destabilized and the single-stranded toehold is available to activate the amplification reaction. As shown in Fig. 6B, the cultured three *Salmonella Typhimurium* were inoculated into PBS with different concentrations from 0 to 8 × 10^7^ CFU mL^−1^ (with 1:4 dilution), respectively. Upon addition of *Salmonella Typhimurium* with increasing concentration, the intensity of absorbance increased successively. The RSD of the intra-assay and inter-assay precisions were 5.0% and 7.6%, respectively. The results indicate that the developed method possesses the potential as a pragmatic tool for *Salmonella Typhimurium* direct detection in real samples. Furthermore, the amplification strategy can be used for detection different targets by means of changing the corresponding complementary target DNA or aptamer strands, demonstrating versatile and potential colorimetric sensing platform.

## Conclusions

In summary, this work proposes a DNA nanotechnology-triggered signal switch and combines the transducer with CHA recycling amplification to develop a highly sensitive and selective strategy for homogeneous visual colorimetric bioanalysis. This strategy has been successfully used for detection of oligonucleotide and *Salmonella Typhimurium*. It can conveniently be extended to a wide range of analytes with available affinity ligands. This developed method shows excellent analytical performance with satisfactory versatility for different analytes. In addition, the proposed strategy possesses good reproducibility and acceptable accuracy for clinical practical sample analysis, and provides a label-free, enzyme-free, low-cost, visual, simple, rapid, sensitive and specific platform for clinical diagnostics.

## Methods

### Measurement Procedures

The experiments were performed in 100 μL of solution containing 50 μL of H0 (400 nM) and 50 μL of target DNA solution of different concentrations. The mixture was first incubated in TNaK buffer for 30 min at 37 °C to allow the hairpin structure to be opened. After that, 50 μL of H1 (500 nM) and 50 μL of H2 (400 nM) was added the above mixture to give the final concentrations of 125 nM and 100 nM, respectively. And further incubation in TNaK buffer for 20 min at 37 °C. Subsequently, 3 μM of hemin was introduced to the resulted solution and incubated for 40 min at room temperature. Finally, 200 uL of ABTS^2−^ (4 mM) and 1 μL of 30% H_2_O_2_ were added to the mixture and mixed completely. The mixture was added into square quartz cuvettes, and the absorption spectra were collected from 500 to 400 nm on a UV-vis spectrophotometer (UV-2550, Shimadzu, Kyoto, Japan). The rate of the peroxidase-mimicking reaction was monitored at 418 nm. All measurements were conducted at room temperature.

## Additional Information

**How to cite this article**: Chen, W. *et al.* DNA transducer-triggered signal switch for visual colorimetric bioanalysis. *Sci. Rep.* **5**, 11190; doi: 10.1038/srep11190 (2015).

## Supplementary Material

Supplementary Information
