# Supplementary material for: DNA transducer-triggered signal switch for visual colorimetric bioanalysis
Source: Sci Rep. 2015 Jun 10;5:11190. doi: 10.1038/srep11190 (PMC4462091; doi:10.1038/srep11190)
Supplement: Supplementary Information [file srep11190-s1.doc]

**Supporting Information**

**DNA transducer-triggered signal switch for visual colorimetric bioanalysis**

Wenhong Chen^a,1^, Yurong Yan ^a,b,1^, Ye Zhang^b^, Xuemei Zhang^b^, Yibing Yin^a,b,*^, Shijia Ding^a,b,*^

^a^ Ministry of Education Key Laboratory of Child Development and Disorders; Key Laboratory of Pediatrics in Chongqing, CSTC2009CA5002; Chongqing International Science and Technology Cooperation Center for Child Development and Disorders; Department of Clinical laboratory, Children’s Hospital of Chongqing Medical University, Chongqing 400014, China

^b^ Key Laboratory of Clinical Laboratory Diagnostics (Ministry of Education), College of Laboratory Medicine, Chongqing Medical University, Chongqing 400016, China

* Corresponding author. Tel: +86-23-68485688; Fax: +86-23-68485786.

E-mail address: [dingshijia@163.com](mailto:dingshijia@163.com) (S.J. Ding) and yibingyin56@126.com

^1^ These authors contributed equally to this work.

**Supplementary** **Reagents and materials.** The gene *lytA* was used to design a specific probe for *S. pneumoniae*, and the primer pairs designed for the obtained sequence were created using the Primer Premier 5.0 software (PREMIER Biosoft International, Palo Alto, CA, USA). The specificity was checked using a Primer-BLAST query in the GenBank database (http://www.ncbi.nlm.nih.gov/BLAST/). All oligonucleotides were synthesized by Sangon Inc. (Shanghai, China) and used without further purification. The sequences of oligonucleotiedes employed in this work are listed in Supplementary Table S3. DNA stock solutions (100 μM) were prepared in TE buffer (10 mM Tris–HCl, pH 8.0; 1 mM ethylenediaminetetraacetic acid (EDTA)). TNaK buffer as the hybridization buffer (pH=7.5) containing 20 mM Tris–HCl, 125 mM NaCl and 20 mM KCl, was stored at 4 ℃ before use. Hemin and 2, 2′-azino-bis (3-ethylbenzothiozoline)-6-sulfonate (ABTS^2−^) were purchased from Sigma-Aldrich (St. Louis, MO, USA). A hemin stock solution was prepared in dimethyl sulfoxide (DMSO) and stored in the dark at −20 °C. TaKaRa PrimeSTAR® HS DNA Polymerase was bought from TaKaRa (Dalian, China). GoldView was purchased from SBS Genetech (Beijing, China). All other reagents were of analytical reagent grade. All aqueous solutions were prepared using Millipore-Q water (≥18 МΩ, Milli-Q, Millipore).

**Bacterial extraction and template preparation.** The bacteria strains employed in this study are listed in Supplementary Table S4. All streptococci were grown in C+Y medium and the others were grown in Luria-Bertani medium at 37 ℃ without shaking until an optical density (at 550 nm) of about 0.5. Viable counts were performed by plating 100 μL of appropriate 10-fold dilutions in sterile PBS solution onto plate count agar in triplicate and incubating the plates for 24 h at 37 ^◦^C. The concentration was estimated by calculating the average number of CFU. The 5 samples of clinical human bronchoalveolar lavage fluid, 5 samples of cerebrospinal fluid that had been previously used for enriched culture and microbiological diagnostic was collected from clinical laboratory of children's hospital of Chongqing Medical University, stored at -80°C until use. The samples were subjected to DNA extraction with the TIANamp Bacteria DNA Kit (TIANGEN, Beijing, China). The sample was eluted in 25 μL of ddH_2_O, which was directly used as PCR template.

**PCR amplification.** PCR was performed in a final volume of 50 μL containing 1.0 μL of 10 μM each primer, 2.0 μL of Prime star polymerase, 2.0 μL of 5×buffer, 2.0 μL of deoxyribonucleotide triphosphate (dNTP), 1.5 μL of genomic DNA, and 20 μL of water. The PCR conditions were: denaturation at 95°C for 5 min, followed by 35 cycles of 95 °C for 1 min (denaturation), 58 °C for 15 sec (annealing), 72 °C for 30 sec (extension), and 5 min final extension. However, as the PCR products were double stranded DNA, which cannot trigger the hybridization, PCR products were first denatured (by heating) into single-stranded DNA (ssDNA) and then hybridized with the hairpin structure DNA.

**Gel Electrophoresis and polyacrylamide gel electrophoresis.** PCR products were identified by running gel electrophoresis in 2.5% agarose gel for 15 min and observed under ultraviolet light. 12% polyacrylamide gels were prepared in 1×TBE buffer. Sample solutions were mixed with 6 × loading buffer and loaded into the gels. The electrophoresis run at a constant voltage of 120 V for about 1 h. The gels were stained with Goldview for 30 min with shaking. Gel images were recorded under UV imaging system (Bio-Rad Laboratories, USA).

**Preparation of Probes.** H1 and H2 were designed referring to our recently published work, based on the principle of the enzyme-free strand-displacement systems. All hairpin probes were heated to 95 °C for 5 min, followed by gradually cooling down to room temperature. Then the obtained DNA solutions were stored at 4 °C for further use.

Table S1. Comparison between the proposed assay and other reported methods for DNA detection

| Analytical technique | Strategy | Detection limit | Reference |
| --- | --- | --- | --- |
| Colorimetry | AuNPs | 0.44 nM | S1 |
| Fluorescence | Exo III and RCA | 3.2 pM | S2 |
| Colorimetry | CHA | 1 nM | S3 |
| Fluorescence | CHA | 0.2 nM | S4 |
| SWV | CHA | 20 pM | S5 |
| Colorimetry | CHA | 32 pM | This work |

Table S2. Assaying results of clinical human bronchoalveolar lavage ﬂuid samples and cerebrospinal fluid samples using colorimetric method and bacterial culture method.

|  | human bronchoalveolar lavage ﬂuid sample | | | | | | human cerebrospinal fluid sample | | | | |
| --- | --- | --- | --- | --- | --- | --- | --- | --- | --- | --- | --- |
| Sample no. | 1 | 2 | 3 | 4 | 5 | 6 | | 7 | 8 | 9 | 10 |
| Colorimetric method (N× 10^3^CFU mL^-1^) | <0.15 | 22.10 | 3.75 | 8.08 | 7.16 | <0.15 | | 8.31 | 3.43 | 2.53 | 2.55 |
| Bacterial culture count(N× 10^3^CFU mL^-1^) | _ | 20.00 | 0.15 | 6.24 | 6.13 | _ | | 6.24 | 0.87 | 1.26 | 1.79 |

-, sterile sample; the absorbance of the sample no. 1 and no. 6 were 0.072 and 0.078, respectively, they were less than detection limit, so < 156 CFU mL^−1^.

Table S3. Oligonucleotides used in the present work.

| Nucleic acid | Sequence (5'- 3') |
| --- | --- |
| T0 | CACACTCAACTGGGAATCCGCATT |
| Hairpin H0 | TGGCAGCCCTTTCTCAATGCGGATTCCCAGTTGAGTGTGAGAAAGG |
| Hairpin H1 | GGGTAGGGCGGGTTGGGATGAGAAAGGGCTGCCACATCCCAACCCATA |
| Hairpin H2 | TATGGGTTGGGATGTGGCAGCCATCCCAAC |
| Primer F | GCAACCATATAGGCAAGTAC |
| Primer R | TTCTGTACGGTTGAATGCGG |
| Apt | TGGCAGCCCTTTCTCTATGGCGGCGTCACCCGACGGGGACTTGACATTATGACAG |
| Inh | CGCCATAGAGAAAGG |
| T1 | CACACTAAACTGGGAATCCGCATT |
| T2 | CACACTAAACTGGGAATCAGCATT |
| NT | ATCGTGTGAACTCGGCTTAATGCC |

Table S4. Bacterial strains used in this study

| Strains | Number | Source or reference |
| --- | --- | --- |
| S. pneumonia D39 | NCTC7466 | National Collection of Type Cultures (London, UK) |
| S. pneumonia 19F | NCTC7466 | National Collection of Type Cultures (London, UK) |
| S. pneumoniae R6 | NCTC7466 | National Collection of Type Cultures (London, UK) |
| S. pneumoniae 6B | NCTC7466 | National Collection of Type Cultures (London, UK) |
| S. pneumoniae TIGR4 | NCTC7466 | National Collection of Type Cultures (London, UK) |
| S. pneumoniae TIGR3 | NCTC7466 | National Collection of Type Cultures (London, UK) |
| S. pneumoniae | ATCC49619 | children's hospital (Chongqing, China) |
| S. mitis | NCTC12261 | children's hospital (Chongqing, China) |
| Streptococcus pyogenes | ATCC19615 | children's hospital (Chongqing, China) |
| Haemophilus influenza | ATCC49247 | children's hospital (Chongqing, China) |
| Staphylococcus aureus | ATCC29213 | American type culture collection (Maryland, USA） |
| Enterococcus faecalis | ATCC29212 | American type culture collection (Maryland, USA） |
| Klebsiella pneumoniae | ATCC700603 | children's hospital (Chongqing, China) |
| E.coli | ATCC25922 | children's hospital (Chongqing, China) |
| Pseudomonas aeruginosa | ATCC27853 | children's hospital (Chongqing, China) |
| Salmonella Typhimurium | ATCC14028 | Chongqing Center for Disease Control and Prevention (Chongqing, China) |

**References**

S1. Luo, R. *et al.* A colorimetric assay method for *invA* gene of *Salmonella* using DNAzyme probe self-assembled gold nanoparticles as single tag. *Sensors and Actuators B.* **198**, 87-93 (2014).

S2. Liu, X. *et al.* A cascade signal amplification strategy for sensitive and label-free DNA detection based on Exo III-catalyzed recycling coupled with rolling circle amplification. *Analyst.* **139**, 2884-2889 (2014).

S3. Ma, C., Wang, W., Li, Z., Gao, L. & Wang, Q. Simple colorimetric DNA detection based on hairpin assembly reaction and target-catalytic circuits for signal amplification. *Anal. Biochem.* **429**, 99-102 (2012).

S4. Li, H., Ren, J., Liu, Y. & Wang, E. Application of DNA machine in amplified DNA detection. *Chem. Commun.* **50**, 704-706 (2014).

S5. Li, B., Ellington, AD. & Chen, X. Rational, modular adaptation of enzyme-free DNA circuits to multiple detection methods. *Nucleic. Acids. Res.* **39**, e110 (2011).
